# Towards harmonizing the concepts and definitions of pain in the World Health Organization's Family of International Classifications

**DOI:** 10.1097/j.pain.0000000000002854

**Published:** 2022-12-22

**Authors:** Michiel F. Reneman, Melissa Selb, Beatrice Korwisi, Antonia Barke, Reuben S. Escorpizo, Samson W. Tu, Rolf-Detlef Treede

**Affiliations:** a Department of Rehabilitation Medicine, University Medical Center Groningen, University of Groningen, Groningen, the Netherlands; b ICF Research Branch, Nottwil, Switzerland; c Swiss Paraplegic Research, Nottwil, Switzerland; d Division of Clinical Psychology and Psychotherapy, Department of Psychology, Philipps-University Marburg, Marburg, Germany; e Division of Clinical Psychology and Intervention, Institute of Psychology, University Duisburg Essen, Essen, Germany; f Department of Rehabilitation and Movement Science, University of Vermont, Burlington, VT, United States; g Center for BioMedical Informatics Research, Stanford University, Stanford, CA, United States; h Mannheim Center for Translational Neuroscience, Medical Faculty Mannheim of Heidelberg University, Mannheim, Germany

## Abstract

Supplemental Digital Content is Available in the Text.

## 1. Introduction

Consistency of language is essential to facilitate high-quality care and patient education, high-quality and generalizable research, and high-quality education for the general public and students of healthcare professions in all stages of academic and professional training.^13^ A uniform language that is universally accepted and understood is key to enhancing communication and targeted delivery of care. The World Health Organization (WHO) has developed a “family” of 3 reference classifications to define and describe diseases, functioning, and interventions: the International Statistical Classification of Diseases and Related Health Problems (ICD),^4,15^ the International Classification of Functioning, Disability and Health (ICF),^20^ and the International Classification of Health Interventions (ICHI).^3,16^ The ICD enables the categorical classification of disease, disorders, and reasons for encounter with the health system, while the ICF provides a framework for documenting functioning and health.^3^ The ICHI is a tool for reporting and analyzing health interventions.^3^ All these classifications are intended for use at the individual and population level.

The ICD, ICF, and ICHI complement each other and can be used jointly,^17^ and consistency of the language across the 3 classifications is critical. To achieve the required consistency among the 3 classifications, their current versions (version 11 for ICD) are fully electronic and are based on the same “Foundation.”^18^ The Foundation is a large collection of all entities that exist in the WHO classification universe. The Foundation contains basic entries for each entity; each entry has to provide a definition and additional information. Table 1 summarizes an overview of the 3 classifications.

**Table 1:** Overview of the World Health Organization Family of International Classifications.

Name	Description	Structure	Common elements
ICD	Classification of diseases, disorders, causes of death, and reasons for encounter with health services	Chapters organised according to body systems and contains related entities such as signs and symptoms	Foundation Maintenance platform
ICF	Framework for measuring functioning and health	Two parts: Part 1: Functioning and disability 1.1. Body functions and body structures 1.2. Activities and participation Part 2: Contextual factors 2.1. Environmental factors
ICHI	Tool for reporting and analysing health interventions	Three axes: T-A-M Target (the entity on which the Action is performed) Action (what is performed by the acting person to a target) Means (the processes/methods by which the Action is performed) A complete ICHI code contains all 3 aspects Codes combine to express coperformed interventions and packages of interventions.

ICD, International Statistical Classification of Diseases and Related Health Problems; ICF, International Classification of Functioning, Disability and Health; ICHI, International Classification of Health Interventions.

These WHO classifications have several different use cases beyond research and clinical care: they are used by governments for resource allocation, by insurances for healthcare reimbursement, and by international organizations for public health initiatives.^17^ For future policy development, the combined use of the ICF and the ICD to document pain, for example, will be a big step forward in providing an evidence-based policy decision on reimbursement, resource allocation, and education in health care. The ICHI, which is currently in the final stage of development, can further support such decision-making with its integration in the diagnosis-related groups (DRGs), which are used for the reimbursement of hospital care in many countries around the globe.

The WHO classifications are applicable for all health conditions and a broad range of functioning entities and interventions. However, they were not specifically designed for pain. The classifications are regularly revised to accommodate advances in knowledge. In 2018, the 11th revision of the ICD was introduced (ICD-11), with major changes in the classification of pain diagnoses vs the 10th revision.^14^ The International Association for the Study of Pain (IASP) had viewed ICD-10 critically for its insufficiencies in describing the representation and disease burden of chronic pain.^9^ Since 2012, the IASP in close dialogue with the WHO has been elaborating on an improved classification of chronic pain^12^ that was integrated into the ICD-11 after a thorough review of corresponding update proposals submitted on the official WHO proposal platform accompanied by supporting evidence from pilot field trials.^1,2,5^ The ICD-11 came into effect in January 2022.^19^

In 2020, IASP initiated a Taskforce (TF) on the “Integration of ICD and ICF” (referred to as “Harmonization TF” from now on), which aims to support the implementation of both ICD-11 and ICF for the purpose of promoting quality care for patients with acute or chronic pain. Because the previous efforts of IASP and the WHO had focused on enhancements in ICD-11 only, the Harmonization TF noticed that there are inconsistencies in concepts and definitions of pain between the ICD-11 and ICF, which are also reflected in ICHI, given that some wording used in the ICF has been adopted in ICHI. For example, the definition of pain in ICHI^16^ corresponds to the definition of pain in the ICF.^20^ To foster the advancement in the field of pain, it is important that the taxonomy used in the WHO classifications is harmonized. Given that all classifications are based on the same Foundation, identifying and resolving such inconsistencies will be paramount. With this white paper, the Harmonization TF reports on the efforts to harmonize the concepts and definitions of pain in ICD-11 and ICF, and consequently also in ICHI, and to further align them with each other and with the broadly accepted multifactorial and biopsychosocial definition of pain by IASP.^6,8^ This white paper also intends to promote public discourse on these important issues, while any resulting revision of the WHO reference classifications is being handled through the official WHO maintenance platform.^21^

## 2. The International Association for the Study of Pain definition of pain

In 1979, the IASP definition of pain^6^ introduced the notion that pain has an affective (or emotional) and a sensory component and, therefore, represents the biopsychosocial definition of pain. People who experience pain generally do not differentiate between the emotional and sensory components. The perception of pain is often induced by potential or actual damage to some body structure: organs or tissues (nociceptive pain) or the somatosensory nervous system (neuropathic pain). However, pain may also be experienced in the absence of any demonstrable bodily damage, such as chronic primary pain conditions (eg, chronic widespread pain, fibromyalgia syndrome, complex regional pain syndrome, chronic low-back pain, or chronic migraine^7^). Pain can also persist beyond the successful healing of tissue damage, as is the case of chronic secondary pain (eg, chronic post-traumatic pain).^11^

The 1979 definition was criticized for not being applicable to preverbal or nonverbal populations such as infants or patients with dementia. These populations can obviously experience acute and chronic pain even if they cannot describe their pain regarding actual or potential tissue damage.^10^ In 2020, the IASP approved the revision of its 1979 definition of pain (Table 2). The current IASP definition of pain is the culmination of a 2-year review process conducted by a 14-member international task force comprising experts in clinical and basic science related to pain. This review process involved a modified Delphi survey of more than 800 persons (clinicians, clinical and basic science researchers, administrators, educators, trainees/students, persons living with pain or with pain-related disability, and caregivers). The current IASP definition of pain (Table 2,^8^) has been included in the ICD-11 (https://icd.who.int/browse11/l-m/en).

**Table 2:** Evolvement of concepts and definitions of pain in the World Health Organization Family of International Classifications.

Source	Definition	Application
IASP 1979	Pain is an unpleasant sensory and emotional experience associated with actual or potential tissue damage or described in terms of such damage	ICD-11 (MG30): before 2021
ICF (b280; current)	Sensation of unpleasant feeling indicating potential or actual damage to some body structure	ICF ICD-11 V chapter ICHI
IASP 2020 (current)	Pain is an unpleasant sensory and emotional experience associated with, or resembling that associated with, actual or potential tissue damage	ICD-11 (MG30): 2021 and later versions ICD-11 V chapter, if the update proposal is accepted ICF (b280; proposed) Future ICHI, if the update proposal is accepted

IASP, International Association for the Study of Pain; ICD, International Statistical Classification of Diseases and Related Health Problems; ICF, International Classification of Functioning, Disability and Health; ICHI, International Classification of Health Interventions.

## 3. Rationale for harmonizing the definitions of pain

Although ICD-11 reflects the internationally accepted multifactorial IASP definition of pain, currently, the ICF does not. The definition of pain in the ICF is described in the entity “b280 Sensation of pain” (Table 2). This ICF entity does not fully reflect the IASP definition because b280 reflects only the sensory component of pain, hence not inclusive. Consequently, it is currently listed in the Sensory functions and pain chapter of the ICF. Thus, the affective (or emotional) component of pain is missing in the ICF. Moreover, pain without potential or actual damage to some body structure (eg, migraine) is also not represented in the ICF, and hence, the ICF does not easily apply to patients with chronic primary pain conditions.

As stated, the ICHI adopted the ICF definition of pain. If the ICF/ICHI definition of pain is applied strictly to pain interventions as listed in the ICHI, patients with chronic primary pain would be excluded from these interventions. Moreover, the multimodal and often interdisciplinary pain treatment concept that also addresses the emotional aspects of pain experience would also not be reflected. Thus, there is an urgent need to harmonize both the ICF and ICHI to the biopsychosocial concept of pain as represented in ICD-11. To meet that need, the Harmonization TF, in consultation with classifications experts, has submitted a proposal for an update to the ICF definition of pain (ie, content enhancement) and to the location of the pain entity in the classification hierarchy (ie, hierarchical change).^21^

## 4. Proposal 1. Content enhancement

The Harmonization TF proposed the following content enhancement of the entity “Sensation of pain”: (b280) in the ICF renaming it as “Pain” with the new description “An unpleasant sensory and emotional experience associated with*, or resembling that associated with*, actual or potential tissue damage.” Likewise, the description of the corresponding subentities would be modified accordingly. For example, generalized pain (b2800) would be *“An unpleasant sensory and emotional experience associated with, or resembling that associated with, actual or potential tissue damage* felt all over, or throughout the body.” Another example, Pain in body part (b2801), would be “*An unpleasant sensory and emotional…*felt in a specific part, or parts, of the body.” The italicized text reflects the proposed enhancement. This updated definition reflects the multifactorial nature of pain and is consistent with the IASP definition of pain^8^. Furthermore, the updated definition will harmonize pain in the ICF with pain in the ICD.

## 5. Proposal 2. Hierarchical change

Pain and its corresponding subentities are inconsistent with the taxonomy of the other entities that are related to the senses (seeing functions, hearing functions, etc.) in chapter 2 of the ICF “Sensory functions and pain.”^20^ The developers of ICHI seemed to have recognized this and decided to establish a separate cluster for interventions for pain instead of keeping it under “Sensory functions.”^16^ Given this, the Harmonization TF proposed a hierarchical change that would involve taking out the whole cluster of pain entities (including corresponding subentities) out of chapter 2 Sensory functions, and pain to become an independent cluster called “Pain and related functions.” As a result, the current chapter 2, Sensory functions, and pain, would be renamed “Sensory functions” and the name “Pain” would replace what is currently called “Sensation of pain.” This proposal is consistent with the content enhancement proposal.

## 6. Implications of the proposed updates

The updates proposed have implications for the ICF and other classifications:

(1) As mentioned earlier, there is similar wording between the ICF and ICHI pertaining to pain; consequently, the content enhancement proposal has implications for the definition of interventions related to pain in the ICHI. If the content enhancement proposal is accepted in the ICF, a revision of the ICHI definitions (Table 3) would then be warranted, but fortunately, its structure will remain.
(2) “Sensation of pain” is also an ICD-11 entity under the Supplementary section on functioning assessment/Generic functioning domains with the same definition as the current ICF definition.^15^ Given that the Generic functioning domains are the same entities in the ICD-11 and ICF, we expect that the proposed hierarchy change, if accepted, will also be reflected in this ICD-11 chapter.
(3) The hierarchical change proposal poses a complex challenge because it may require the creation of new codes in the ICF for the pain entities that reflect where in the classification the proposed new cluster is placed after disentangling Pain from chapter 2 Sensory functions and pain. If the new cluster is placed immediately after the renamed chapter 2 “Sensory functions,” the question is whether it is appropriate to renumber the current chapters 3 to 8 and their respective entities because the new cluster Pain may take over chapter 3 and its entities would take over the codes b310, b320, b330, etc. from the current chapter 3 “Voice and speech functions” to follow the consecutive sequencing of chapters and codes of the classification system. Such an approach of reusing codes for different concepts is generally unacceptable in standard terminologies and classifications because it poses continuity problems between earlier and later versions of the same classification. However, if the new pain cluster is moved to the end as an additional chapter, ie, chapter 9 Pain, then only new codes for pain entities (b910, b920, etc.) would be necessary rather than having to renumber current chapters 3 to 8. Given the close relationship between pain and sensory functions, we believe that it would be ideal to place the new cluster immediately after the “Sensory function” cluster, provided an acceptable solution can be found for the coding problem stated earlier.

**Table 3:** Pain interventions as currently listed in the International Classification of Health Interventions, modified wording according to proposed wording change in the International Classification of Functioning, Disability, and Health.

ICHI code	Name of the pain intervention	Current definition	Proposed new definition
AXA.AA.ZZ	Assessment of pain	Evaluating sensation of unpleasant feeling indicating potential or actual damage to some body structure	Evaluating unpleasant sensory and emotional experience associated with, or resembling that associated with, actual or potential tissue damage
AXA.AC.ZZ	Test of pain	Using a questionnaire, rating scale or other instrument to test sensation of unpleasant feeling indicating potential or actual damage to some body structure	Using a questionnaire, rating scale or other instrument to test unpleasant sensory and emotional experience associated with, or resembling that associated with, actual or potential tissue damage
AXA.DB.AC*	Oral or enteral medication for pain	Administration of medication by oral or enteral route	Unchanged
AXA.DB.AF	Parenteral medication for pain	Administration of medication by injection	Unchanged
AXA.DB.AH	Transdermal medication for pain	Administration of medication by transdermal patch	Unchanged
AXA.DB.ZZ	Pharmacotherapy for pain, not elsewhere classified	Administering a pharmaceutical substance to assist with managing pain	Unchanged
AXA.PG.ZZ	Assisting and leading exercise for pain	Supporting or guiding exercise focusing on functions related to sensation of unpleasant feeling indicating potential or actual damage to some body structure	Supporting or guiding exercise focusing on functions related to unpleasant sensory and emotional experience associated with, or resembling that associated with, actual or potential tissue damage
AXA.PM.ZZ	Education about pain	Providing information to improve knowledge about sensation of unpleasant feeling indicating potential or actual damage to some body structure	Providing information to improve knowledge about unpleasant sensory and emotional experience associated with, or resembling that associated with, actual or potential tissue damage
AXA.PN.ZZ	Advising about pain	Providing advice to encourage a change of or to maintain sensation of unpleasant feeling indicating potential or actual damage to some body structure in relation to health (or risks)	Providing advice to encourage a change of or to maintain unpleasant sensory and emotional experience associated with, or resembling that associated with, actual or potential tissue damage
AXA.PP.ZZ	Counselling for pain	Providing therapeutic and/or supportive communication in relation to functions of sensation of unpleasant feeling indicating potential or actual damage to some body structure	Providing therapeutic and/or supportive communication in relation to functions of unpleasant sensory and emotional experience associated with, or resembling that associated with, actual or potential tissue damage
AXA.PQ.ZZ	Psychotherapy for pain	Providing therapeutic communication, based upon the systematic application of psychological theory, in relation to sensation of unpleasant feeling indicating potential or actual damage to some body structure.	Providing therapeutic communication, based on the systematic application of psychological theory, in relation to unpleasant sensory and emotional experience associated with, or resembling that associated with, actual or potential tissue damage
AXA.SC.BP	Electrical stimulation for pain	Provoking or inciting a reaction by application of electric current in relation to sensation of unpleasant feeling indicating potential or actual damage to some body structure	Provoking or inciting a reaction by application of electric current in relation to unpleasant sensory and emotional experience associated with, or resembling that associated with, actual or potential tissue damage
AXA.SE.ZZ	Hypothermy for pain	Applying cold to the body or part of the body in relation to sensation of unpleasant feeling indicating potential or actual damage to some body structure	Applying cold to the body or part of the body in relation to unpleasant sensory and emotional experience associated with, or resembling that associated with, actual or potential tissue damage
AXA.ZZ.ZZ	Other interventions for pain, not elsewhere classified	Other interventions related to the sensation of pain	Other interventions related to modifying the experience of pain

ICHI, International Classification of Health Interventions. ICF, International Classification of Functioning, Disability and Health

* The code reflects the 3 axes (cf Table 1): AXA (target: pain); DB (action: administering medication); AC (means: by oral or enteral route). It should be noted that this list does not aim at being exhaustive. Many interventions that can also be applied for pain are found in different ICHI chapters (eg, surgical interventions in the chapters for the respective body parts). Users of the ICHI can also combine intervention codes themselves based on the 3 axes.

## 7. Conclusion

The WHO emphasizes that its reference classifications ICD-11, ICF, and ICHI share a common terminological foundation, which facilitates the joint use of these classifications, uniform communication among various stakeholders, and comparable data transfer, among other things.^17^ The proposal by the Harmonization TF to update the definition of pain in the ICF as described in this white paper will further strengthen the common terminological foundation across all WHO classifications in the field of pain, while also aligning with the internationally accepted multifactorial and biopsychosocial IASP definition of pain^8^ already reflected in the ICD-11. The harmonization of the concepts and definition of pain in the WHO reference classifications is expected to ultimately improve access to multimodal and interdisciplinary pain interventions and support high-quality pain management for patients.

## Conflict of interest statement

The authors have no conflict of interest to declare.

## Supplemental video content

A video abstract associated with this article can be found at http://links.lww.com/PAIN/B767.
